# Genetic Testing for *GBA* and *LRRK2* Mutations: Is it Time for Routine Use?

**DOI:** 10.1002/mdc3.13619

**Published:** 2023-01-20

**Authors:** Karisha Roopnarain, Christine Klein

**Affiliations:** ^1^ Institute of Neurogenetics University of Luebeck and University Hospital Schleswig‐Holstein Luebeck Germany; ^2^ Faculty of Medicine and Health Sciences Stellenbosch University Cape Town South Africa

**Keywords:** Parkinson's disease, *LRRK2* pathogenic variants, *GBA* pathogenic variants, Parkinson's disease genetic etiology, genetic testing for Parkinson's disease, monogenic Parkinson's disease

In 1997, Mihael H. Polymeropoulos and colleagues announced to the world that a mutation in the α‐synuclein (*SNCA*) gene was identified in families with Parkinson's disease (PD).^1^ This discovery not only confirmed the role of genetics in the etiology of PD but also set the stage for future research in the field of genetics and underlying pathophysiological mechanisms in PD. Twenty‐five years later, we have made many breakthroughs in understanding the genetics of PD and the mechanisms they bring about disease, which has led to the development of the first gene‐targeted therapies. We are now in an exciting era, where several of these therapies are entering clinical trials, taking us a step closer to finally being able to offer our patients with PD hope of a causal treatment. Should these medical advancements prompt us clinicians taking care of patients with PD to offer routine genetic testing for glucocerebrosidase (*GBA*) and leucine‐rich repeat kinase (*LRRK2*) pathogenic variants, as they are the most commonly known PD risk and causal variants and the most targeted in gene‐specific interventional trials?

At first sight, the answer to this question would be a no, because although there are various clinical trials ongoing, we currently have no US Food and Drug Administration (FDA)–approved gene‐targeted therapy available, so genetic testing in the present climate would not affect management. However, in certain circumstances, our answer would be different, and we would offer genetic testing to our patients with PD. These circumstances would include an uncertain diagnosis with atypical features before more invasive investigations are considered, an earlier age of onset to help with prognosis, and counseling of patients and their families about disease progression, patients' interests, and inclusion in clinical trials.

## Worldwide Mutation Frequencies of *GBA* and *LRRK2*

Pathogenic variants in the *GBA* and *LRRK2* genes together account for most of the known strong genetic contributions to PD, with variants in *LRRK2* being the most frequent cause^2^ with an average mutation frequency of 3.1%^3^ and variants in *GBA* being the most frequent risk factor^4^ with a frequency of pathogenic variants of 8.5%.^3^ However, the frequency of these variants differs in different ethnic groups. The G2019S variant is the most frequent *LRRK2* pathogenic variant: It is found in 10% of familial PD cases, 1% to 2% of all PD cases,^5^ 3% in Europeans, 16% to 19% in Ashkenazi Jews, and up to 42% in African Arab‐Berbers^6^ and in most other populations investigated with the exception of several Asian populations.^7^ Pathogenic *GBA* variants have been identified in various populations but are most frequently detected among people of Ashkenazi Jewish descent.^8^ *LRKK2* and *GBA* mutations bridge the etiological gap between sporadic and hereditary PD, being both risk and causal factors to variable degrees on a continuum of reduced penetrance of a causative variant and increased risk. This phenomenon has been confirmed in genome‐wide association studies, cumulative burden tests, and in family studies for *LRRK2*.^9^ *GBA‐PD* is a non‐Mendelian form of PD, but *GBA* mutations markedly increase the risk of developing PD.^10^ A meta‐analysis showed that there was a significant differential effect of severe versus mild *GBA* mutations on the risk and age at onset (AAO) of PD, and the odds ratio for PD ranged between 2.84 and 4.94 for mild *GBA* mutations and 9.92 and 21.29 for severe *GBA* mutations with a 5‐year earlier AAO for severe mutations.^11^

## Penetrance and Risk

“What is my risk of developing PD?” asked a young, healthy, asymptomatic lady who tested positive for a pathogenic *LRRK2* variant and whose father, with PD, had tested positive earlier for this variant. The incomplete penetrance and degree of conferred risk of PD in *LRRK2* and *GBA* mutation carriers make it difficult to predict the development of disease in asymptomatic carriers and should be considered when counseling patients with PD and their families. When neuroprotective therapies become available, we must learn more about the penetrance of PD in these mutation carriers and their predictive value. The only way we can achieve this goal is through widespread genotyping and deep phenotyping of patients with PD and the investigation of genetic modifiers. The French PD Genetic Study group estimated that PD penetrance in *GBA* mutation carriers was 29.7% at 80 years using a dominant model,^12^ which is strikingly similar to the penetrance of the *LRRK2* G2019S variant^13^ and may posit *GBA* as an autosomal dominant condition with reduced penetrance.

There are 2 groups to consider when discussing routine genetic testing: people with PD and people at risk of developing PD with a positive family history. In both groups, we would argue against routine testing, and this will only change if gene‐targeted therapy is available in the PD group and if neuroprotective treatment is available in the group at risk for PD. With this in mind, are there any benefits, and what are the drawbacks of testing unaffected family members? The knowledge of one's mutation carrier status can assist with reproductive decisions, counseling about their risk of disease development, and the identification of individuals for neuroprotective trials when they are available. But this will come at a price, as insurance may not cover the costs of genetic testing and counseling as the result may not be an actionable finding. The other important drawbacks of genetic testing in this group are the negative social, psychological, and economic implications, as asymptomatic mutation carriers may be denied life insurance or future employment opportunities.

## Clinical Clues and Consequences

Are there clinical clues that can distinguish *LRRK2‐PD* and *GBA‐PD* from idiopathic PD (iPD) ?

*LRRK2‐PD* ^14^ (*MDS* gene) and *GBA‐PD* ^15^ can present very similar to iPD except for a slightly earlier AAO in some patients, with mean AAOs of 59.4 years in *LRRK2* variant carriers and 55.8 years in *GBA* variant carriers.^3^ However, with *GBA‐PD*, the phenotype is largely dependent on the severity of the variant type, with severe variant carriers displaying a more severe phenotype than mild variant carriers and iPD. This severe phenotype in *GBA‐PD* includes an earlier AAO, more severe cognitive decline, more frequently developed Lewy body dementia, rapid motor progression, and reduced survival.^16^

Are there important clinical consequences of *LRRK2‐PD* and *GBA‐PD* that would make routine genetic testing justifiable? Because *LRRK2‐PD* and *GBA‐PD* can look similar to iPD and their presentation can be equally heterogeneous, no single clinical sign can adequately indicate a particular gene mutation. Thus, the only way to definitively rule out or rule in a genetic cause is by genetic testing, and this is also the only strategy to identify participants for clinical trials. Consider the following scenario: a 50‐year‐old man presents to a neurology clinic, and he has mild motor features of PD with normal cognition and a negative family history. He could have an *LRRK2* or *GBA* pathogenic variant or just have iPD. Although routine testing for *LRRK2* pathogenic variants does not significantly alter clinical management, testing for the type of *GBA* variant a patient has can affect the way we plan management and counsel the patients and their families already today. A patient diagnosed with *GBA‐PD* is often afraid of developing dementia; however, the risk of dementia and development of Lewy body dementia is higher with severe *GBA* mutations,^17^ but even then, the patient may not develop cognitive impairment. On the other hand, many patients with iPD eventually develop dementia,^18^ but carrying a severe *GBA* mutation does put the patient in a higher risk group for developing dementia, and genetic testing may be beneficial in these cases to better inform counseling and prognosis despite the aforementioned uncertainties in an individual patient. In the same way, there is evidence that shows cognitive impairment worsened after deep brain stimulation (DBS) in patients with *GBA* mutations,^19^, ^20^, ^21^ but many of these studies involved a small number of *GBA* carriers and did not always specify which *GBA* variant was associated with this outcome. Therefore, the detection of a *GBA* mutation in a patient being considered for DBS should not be used as a strict exclusion criterion, and more large‐scale studies investigating the differential effect of *GBA* mutation types and DBS are needed before a firm conclusion can be drawn about DBS in these patients.

## Gene‐Targeted Therapies

Insight into the pathophysiological pathways involved in the genetic forms of PD^2^ can provide important therapeutic targets for gene‐targeted therapies. Of all the monogenic forms of PD, *LRRK2* and *GBA* mutations are the most advanced targets in several trials because they are considerably more common than the other monogenic forms of PD attributed to *SNCA, PINK1*, Parkin, and *DJ1* mutations, for which it will be difficult to achieve an adequately powered clinical trial.^22^ Widespread genetic testing of patients with PD can help stratify appropriate candidates for these gene‐targeted trials. Currently, this is only possible in the realm of large research projects such as the Global Genetics Parkinson Project (GP2) Monogenic hub^23^ that will identify monogenic forms of PD globally, the PD GENEration study (https://www.parkinson.org/PDGENEration; NCT04057794) that provides free CLIA‐certified genetic testing and counseling to patients with PD, or industry‐sponsored interventional studies^3^ that offer genetic testing to prospective participants. The best time to test disease‐modifying treatments would be in presymptomatic mutation carriers; however, this would entail screening the general population, which would be a major challenge.^2^ One way around this would be to test and include relatives of *LRRK2* and *GBA‐PD* in clinical trials, but the incomplete and variable penetrance of these mutations would also pose a major hurdle.

There are 2 main gene‐targeted approaches to *LRRK2*. *LRRK2‐PD* pathogenic variants cause a toxic gain‐of‐function increase in *LRRK*2 kinase activity, which has been targeted by the development of kinase inhibitors.^2^ A total of 2 *LRRK2* kinase inhibitors, DNL201 (NCT03710707) and DNL151 (NCT04056689), will soon enter late‐stage clinical trials, with DNL151 being advanced first (Denali Therapeutics, South San Francisco, CA). There are other inhibitors in preclinical phases and include MLi‐2, PF‐06685360,^24^ and EB‐42168, a selective kinase inhibitor that may avoid the peripheral toxic effects of the nonselective inhibitors.^25^ The second approach is antisense oligonucleotides (ASOs) to reduce *LRRK2* mRNA transmission. A mouse PD model^26^ demonstrated that *LRRK2* ASOs injected into the brain reduce *LRRK2* protein levels and the formation of α‐synuclein inclusions. A phase I trial is underway to assess the safety, tolerability, and pharmacokinetics of intrathecal administration of an ASO called BIIB094 that targets *LRRK2* mRNA expression in patients with PD (clinicaltrials.gov, NCT03976349). For *GBA‐PD*, there are 3 gene‐targeted approaches.^27^ The first approach is glucocerebrosidase substrate reduction using the glucosylceramide synthase inhibitor, venglusat, to decrease α‐synuclein expression (MOVES‐PD). The second approach is gene therapy via a viral vector that can penetrate the blood–brain barrier and increase Glucocerebrosidase (GCase) levels in the brain (AAVa‐PR001A). The third approach enhances GCase activity in the brain by using a repurposed drug called ambroxol (NCT02941822, NCT02914366), a mucolytic used for cough. A small, uncontrolled study confirmed the safety, tolerability, and ability of ambroxol to penetrate the cerebrospinal fluid (CSF) of patients with PD and increase CSF GCase protein levels in patients with PD with a *GBA* pathogenic variant and those without.^28^ Important challenges for these genotype‐driven trials are the lack of routine genetic testing, problems with interpreting the test results, lack of availability of biomarkers to guide treatment responses, and comedication with dopaminergic agents.^29^

## Clinicians' Versus Patients' Views

With the advent of gene‐specific clinical trials, are clinicians and patients with PD and their families ready for routine genetic testing as a standard of care, and are their knowledge, interests, and attitudes toward genetic testing in PD aligned? In 2019, 178 movement disorder specialists at 146 clinical sites in Canada and the United States completed a questionnaire evaluating their current practice, knowledge, attitudes, and barriers to genetic testing in PD.^30^ Of the respondents, 41% had not referred any PD patient for genetic testing, and the most common reason included cost or lack of insurance coverage, lack of perceived clinical utility, and lack of confidence in their genetic knowledge. These are major barriers that need to be overcome before genetic testing is routine in patients with PD. When clinicians’ attitudes toward general screening of the Ashkenazi Jewish population for the breast cancer (*BRCA1/2*) gene and *LRRK2* mutations were compared,^31^ 52% were in favor of *BRCA* screening, being an actionable finding, but 86% opposed *LRRK2* screening, which currently has no specific treatment available. However, this will likely change should a gene‐targeted therapy for *LRRK2* become available and may even have a disease‐modifying effect. Genetic testing raises social and ethical concerns. Many clinicians are concerned about the implications of the genetic test results for their patients and families and need to consider their patients' attitudes and beliefs,^30^ including any potential stigma attached to a genetic diagnosis. But are patients with PD interested in genetic testing? A study in the United States assessing the knowledge, attitudes, and interests of patients with PD in genetic testing^32^ found that they were interested in genetics and genetic testing but lacked the associated genetics knowledge and overestimated their risk. Another study compared the knowledge and attitudes toward genetic testing in PD in American and Asian populations^33^ and reported that the American patients with PD had a higher level of knowledge of PD genetics, and this was associated with a more positive attitude toward genetic testing. Hence, an essential step in preparation for routine genetic testing in PD is to improve the genetics knowledge of both clinicians and patients.

## The Choice and Cost of Genetic Testing and Who Should Pay for It

Genetic tests available for PD include targeted testing in which a single or few variants in (a) gene(s) are tested, multigene panels in which multiple PD genes are tested by next‐generation sequencing together with deletion/duplication analysis offered by commercial companies, and direct‐to‐consumer testing.^34^ However, few laboratories have testing for *LRRK2* and *GBA* mutations on the same panel. A potential solution would be to create a high‐quality sequencing panel covering both the *LRRK2* and *GBA* genes that is easily accessible and low cost.

As genetic testing is evolving, the costs are declining. In countries where health insurance is standard, different insurance companies offer different coverage depending on a patient's/relative's personal risk and family history. Genetic testing and counseling are billed separately, so overall costs are greater with counseling included. Patients or relatives may have to pay out of pocket or get payment plans with the genetic laboratories. When gene‐targeted therapy is available, insurance companies will have an incentive to pay for genetic testing as there will be an actionable finding. There are a few research studies^34^ that offer free genetic testing ± counseling to patients with PD, such as Parkinson's Progression Markers Initiative and PDGENEration (Parkinson's Disease Genetic registry), sponsored by The Michael J. Fox Foundation and the Parkinson's Foundation, respectively. In addition, several pharmaceutic or genetic testing companies offer free genetic testing to patients with PD with no obligation to commit to the ensuing trial. A combination of industry and research would be the best option for the payment of genetic testing to patients with PD and populations where these clinical trials are available.

In the hypothetical situation that a gene‐targeted therapy is FDA approved and available, how would our approach to genetic testing in PD change? We would recommend initiating genetic testing using a stepwise approach, where we first test patients for the specific pathogenic variant(s) or gene(s) that the therapy is directed against and where this therapy is available. Table 1 compares the opportunities and challenges of genetic testing in the clinical versus research setting and when gene‐targeted treatment is available. At present, we are just seeing the tip of the iceberg of patients with PD with a genetic cause, whereas the vast majority of patients do not have access to genetic testing, so the genetic diagnoses of many patients are buried. The more imminent future may hold gene‐targeted treatments that may also become an option for neuroprotective treatment in the more distant future, each requiring different testing approaches. As gene‐targeted therapies become available, genetic testing may become routine in patients with PD, but it is likely that these treatments will be expensive and accessible to only a minority of patients with PD, and we will have to overcome the same problem as with access to genetic testing.

**TABLE 1:** *Comparison of the opportunities and challenges of genetic testing for* GBA *and* LRRK2 *mutations in clinical versus research settings and when gene‐targeted treatment becomes available*

Opportunities	Challenges
Clinical setting	
1. Resolve uncertainty in patients with atypical presentations	1. Majority of patients do not have access to genetic testing
2. Assist in counseling patients about their prognosis and clinical course	2. Shortage of genetic counselors and clinicians with adequate backgrounds
3. Enable testing and counseling of family members about their risk	3. Incomplete penetrance difficult to predict disease and no neuroprotective treatment
4. Plan management, especially in *GBA* severe mutation carriers	4. Specific counseling of patients according to gene and mutation/pathogenic variant involved
5. Patients' interest in knowing diagnosis	5. Costly and insurance may not cover it, and positive results may have negative social and economic implications for patients
6. Will increase clinicians' interest and awareness of genetic diseases	6. Does not alter management much; no clinical utility currently
Research setting	
1. Identify participants for clinical trials	1. Many patients may not have access to studies
2. Identify presymptomatic individuals for neuroprotective trials	2. No neuroprotective treatments currently available
3. Uncover genetic diagnoses	3. Patient may not get back the results as genetic testing within research projects is often not as comprehensive compared with a next‐generation sequencing panel, and they often do not include genetic counseling
4. Investigate genetic modifiers of penetrance	
5. Determine the worldwide frequency of variants	
6. Enable detailed genotype–phenotype correlations	
When gene‐targeted treatment is available	
1. Identify patients who will benefit from this treatment	1. Treatment is likely to be costly, and many patients may not be able to afford it
2. Able to offer patients a disease‐modifying treatment option and hope	2. Access to genetic testing may still be a problem in certain regions
3. Identify presymptomatic individuals in case these treatments can also be used for neuroprotection	3. Demand may increase rapidly without a parallel increase in genetic counselors
4. Will encourage patients and clinicians to improve their genetic knowledge	4. Patients and doctors may not be ready
5. Clinicians more likely to offer genetic testing to their patients	
6. Insurance companies may cover testing as actionable finding	

Abbreviations: *GBA*, glucocerebrosidase; *LRRK2*, leucine‐rich repeat kinase.

In conclusion, the multitude of clinical trials ongoing infuses hope of a gene‐targeted therapy being available to patients with PD soon. However, we do not feel it is time to offer genetic testing routinely until such gene‐targeted treatment is available to patients with PD and neuroprotective therapy is available to asymptomatic mutation carriers. We would recommend genetic testing in patients with PD with atypical features, early age of onset, significant family history, and in the research setting for inclusion in clinical trials.

## Author Roles

(1) Manuscript: A. Writing of the First Draft, B. Review and Critique.

K.R.: 1A

C.K.: 1B

## Disclosures

**Ethical Compliance Statement:** The authors confirm that the approval of an institutional review board and patient consent was not required for this work. We confirm that we have read the Journal's position on issues involved in ethical publication and affirm that this work is consistent with those guidelines.

**Funding Sources and Conflict of Interest:** Christine Klein has received grants from the German Research Foundation, Bundesministerium für Bildung und Forschung (BMBF), The Michael J. Fox Foundation, and Aligning Science Across Parkinson's (ASAP); is employed by the University of Luebeck and University Hospital Schleswig‐Holstein; has received speaking honoraria from Desitin; provides consultancies for Centogene and Lundbeck; has received royalties from Oxford University Press; and is on the advisory board of Retromer Therapeutics. K.R. is a PhD student at the University of Luebeck and University Hospital Schleswig‐Holstein, has received a PhD scholarship from ASAP, and has no conflicts of interest.

**Financial Disclosures for the Previous 12 Months:** Christine Klein would like to acknowledge support from Deutsche Forschungsgemeinschaft (DFG) FOR 2488 and the Global Parkinson's Disease Genetics Program (GP2) funded by the Aligning Science Across Parkinson's initiative and implemented by The Michael J. Fox Foundation. Karisha Roopnarain would like to acknowledge support from the GP2 funded by the Aligning Science Across Parkinson's initiative and implemented by The Michael J. Fox Foundation.
